# Lady Roberts' Field Glass Fund

**Published:** 1917-12-15

**Authors:** John Penoyre


### Lady Roberts' Field Glass Fund.

*To the Editor of* The Hospital.

Sir,—With a restricted issue your readers will excuse the shortest possible letter, and it will be good of you to find room for that.

This Fund, Lord Roberts' last inspiration for the Army, has now issued 30,000 instruments, and has no funds beyond the sum necessary for returning the glasses to their owners in accordance with his wish. Our main expense is repairing the glasses that come back to us for reissue.

We could carry on and meet our repairing bills with one thousand pounds. I am in a position to say that the need justifies the request. May I, therefore, make it?

Yours faithfully,

December 11, 1917. John Penoyre.

Address for sending cheques (also any field glasses and telescopes that can still be spared): The Manager, Lady Roberts' Field Glass Fund, 64 Victoria Street, S.W. 1.

